# Agreement of IVC Diameter Measurements via Subcostal and Transhepatic POCUS Views

**DOI:** 10.24908/pocusj.v10i02.18823

**Published:** 2025-11-17

**Authors:** Santiago Beltramino, Giuliano Gaudenzi, Jhon Mauricio Rojas, Agustín Manchado Bruno

**Affiliations:** Instituto de Trasplante y Alta Complejidad, Buenos Aires, Argentina

**Keywords:** Cava vein, Shock, Fluids tolerance, Dysnea

## Abstract

**Background:**

Inferior vena cava (IVC) point of care ultrasound (POCUS) is essential for hemodynamic evaluation, with the subcostal view (SCV) being the gold standard. However, in situations where this view is inaccessible, the transhepatic view (THV) is a viable alternative. This study evaluates the concordance between these two views in ventilated and non-ventilated patients, categorizing the IVC as small, intermediate, or large.

**Methods:**

This prospective observational study included 99 patients; healthy volunteers, ventilated patients, and non-ventilated patients. We measured end-expiratory IVC diameter, classified as small (<10 mm), intermediate (10–20 mm) or large (>20 mm), via SVC and THV. We then assessed agreement by categorical concordance, using Bland–Altman (mean bias ± 95% limits) and Pearson's correlation (r).

**Results:**

The overall concordance between both views was 83.8% (83/99; 95% CI: 76.4–90%). By IVC diameter category, concordance was 93.8% (15/16; 95% CI: 69.8–99.8) for small, 84% (42/50; 95% CI: 70–90 %) for intermediate, and 82% (27/33; 95% CI: 77–95%) for large. Concordance was unaffected by ventilation status (p = 0.83), but patients with Body Mass Index (BMI) ≥ 30 had lower concordance than those with BMI < 30 (73.9% vs. 89.5%; p = 0.086). The Bland–Altman analysis showed a mean bias of +0.22 mm with 95% limits of agreement from –6.99 to +7.43 mm. Pearson's correlation coefficient for the 99 paired measurements was r = 0.86 (p < 0.001), overall, and when stratified by category was r = 0.81 (small), r = 0.78 (intermediate) and r = 0.74 (large) (all p < 0.001). The sensitivity and specificity of THV for identifying “responders” (CI > 42%) were 28% and 93%, respectively.

**Conclusion:**

The THV is a reliable alternative for categorical evaluation of the IVC, particularly when the SCV is inaccessible. This method supports rapid and accurate clinical decision, especially for dichotomous POCUS decisions but should be used cautiously in patients with elevated BMI.

## Introduction

Inferior vena cava (IVC) ultrasound is a crucial tool for hemodynamic assessment, offering key information about intravascular volume and right atrial pressure (RAP). The subcostal view (SCV), considered the reference approach, provides direct and precise visualization of the IVC in its longitudinal section which enables accurate measurement. Current guidelines recommend using the IVC diameter in SCV combined with the collapsibility index (CI-IVC) during the respiratory cycle to estimate RAP [1].

In certain clinical scenarios, such as postoperative states, abdominal distension, pregnancy, or obesity, the SCV may be limited or inaccessible. For instance, up to 20% of pregnant patients cannot achieve adequate visualization [2]. In these cases, the transhepatic view (THV) has emerged as a potentially viable alternative. Previous studies have primarily focused on the correlation between SCV and THV; specifically, the linear relationship between their diameter measurements and have reported mixed results [3,4]. However, correlation alone does not address whether both views classify the IVC into the same clinically meaningful size categories (small, intermediate, large). Concordance, which quantifies the proportion of exact categorical matches, offers a more applicable metric for point of care ultrasound (POCUS) guided decision-making but remains poorly explored in this context.

Categorizing the IVC (small, intermediate, or large) has proven to be a practical approach for guiding rapid decision-making in both critical and routine situations [5]. The primary aim of this study was to evaluate the concordance between IVC maximum diameter measurements from SCVs and THVs—both overall and within specific patient groups of healthy volunteers, ventilated and non-ventilated patients—to determine whether the axillary view could serve as a viable alternative in certain clinical scenarios.

## Methods

### Study Design and Objectives

This prospective observational study was conducted at the Institute of Transplantation and High Complexity (ITAC) in Buenos Aires, Argentina, between October and December 2024. The primary objective was to assess categorical concordance and linear association (Pearson's r) between maximum end-expiratory IVC diameters measured by SCVs and THVs, classifying the IVC as small (< 10 mm), intermediate (10–20 mm), or large (> 20 mm). The secondary objective was to compare the CI-IVC between SCV and THV for identifying fluid-responsive patients (CI-IVC > 42%).

### Study Population

Inclusion Criteria: Adults (≥ 18 years) with both SCVs and THVs yielding clear, well-defined IVC images suitable for diameter measurement.

Exclusion Criteria: IVC thrombosis or congenital malformations of the IVC were present.

### Procedure

**Figure 1.**
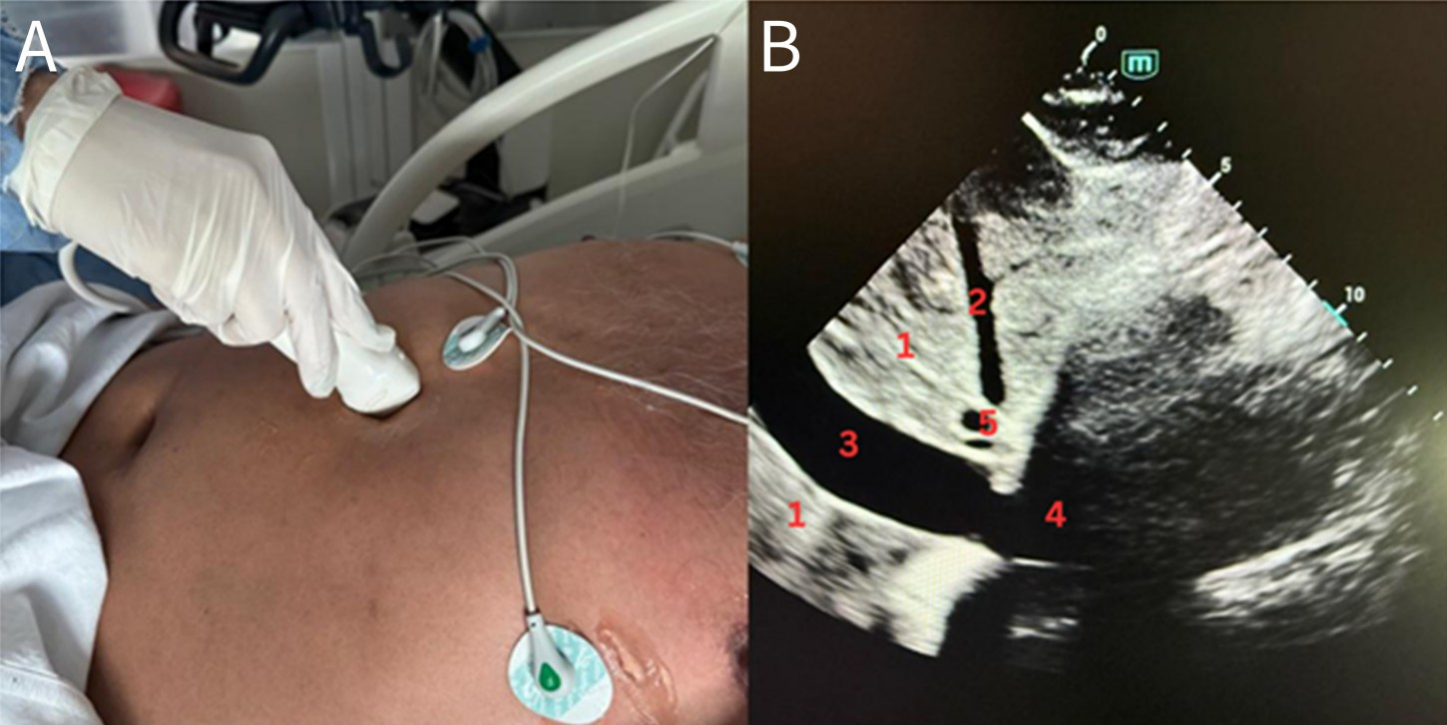
A) Subcostal view (SCV): Transducer placed at the epigastrium in a longitudinal orientation. B) Structures identified are 1. Liver; 2. Middle hepatic vein; 3. Inferior vena cava (IVC) “large”; 4. Right atrium; 5. Portal vein.

**Figure 2.**
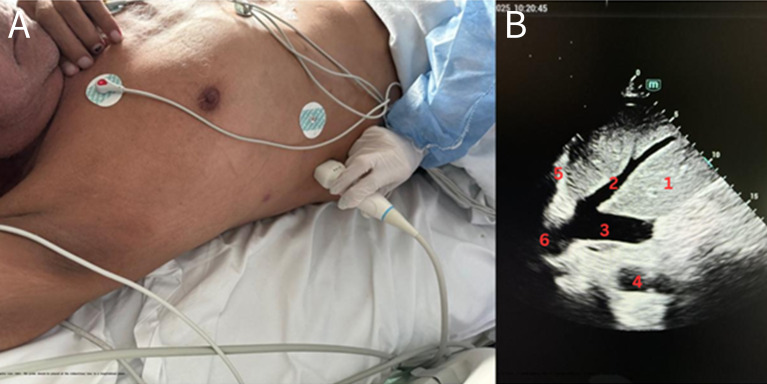
A) Transhepatic view (THV): Transducer placed at the mid-axillary line in longitudinal orientation. B) Structures identified are 1. Liver; 2. Middle hepatic vein; 3. Inferior vena cava (IVC) “large”; 4. Aorta; 5. Diaphragm; 6. Right atrium.

**Figure 3.**
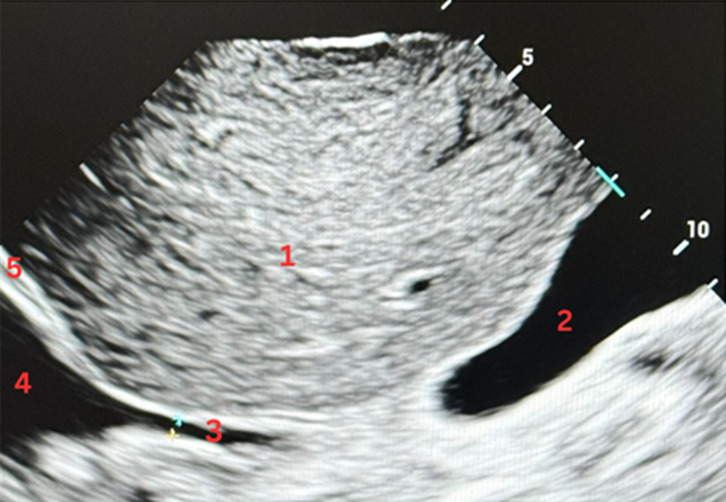
Transhepatic view (THV) in a patient with cirrhosis. The inferior vena cava (IVC) appears small. Structures identified are 1. Liver, 2. Gallbladder, 3. Inferior vena cava (IVC), 4. Right atrium, 5. Diaphragm.

Ultrasound examinations were conducted at the patient's bedside using a Mindray with a 2.5–5 MHz sectorial transducer. Each patient was first scanned in SCV (Figure 1) and immediately thereafter in THV (Figure 2). Both views were in the longitudinal plane during quiet respiration. Three experienced POCUS operators, each responsible for a distinct subgroup of patients, acquired and digitally archived the cine loops. A fourth ultrasound expert then reviewed all stored images offline. For each view, the maximum IVC diameter at end-expiration and at end-inspiration was measured across three consecutive respiratory cycles, and the mean of those measurements was taken as the definitive diameter. Diameters were classified as small (< 10 mm) (Figure 3), intermediate (10–20 mm), or large (> 20 mm).

### Variables Measured

Continuous measurements included (1) categorical concordance, defined as the percentage of cases in which the SCV and the THV assigned the same IVC size category; (2) linear association, expressed by Pearson's correlation coefficient (r) between the continuous diameters from both views; and (3) numerical agreement, assessed by the mean bias and 95% limits of agreement in Bland–Altman analysis. Finally, we measured dynamic respiratory change via the CI-IVC, calculated as (maximum expiratory diameter − minimum inspiratory diameter)/maximum expiratory diameter × 100, to capture the percentage reduction in IVC diameter during inspiration.

### Statistical Analysis

All categorical concordance estimates were accompanied by exact Clopper–Pearson 95% confidence intervals. To assess the strength and significance of the linear relationship between continuous SCV and THV measurements, we calculated Pearson's correlation coefficient along with two-tailed p-values. Numerical agreement was evaluated via Bland–Altman plots, reporting the mean bias and the 95% limits of agreement. We also conducted predefined subgroup analyses comparing mechanically ventilated vs. spontaneously breathing patients and those with BMI < 30 kg/m^2^ vs. BMI ≥ 30 kg/m^2^. All statistical computations were performed using R version 4.2.0 and SPSS version 27.

### Sample Size

An initial calculation (anticipated r = 0.7; 80% power; α = 0.05) yielded 50 patients. We increased our enrollment to 99 patients to ensure balanced representation across IVC categories and clinical subgroups (healthy volunteers, ventilated, non-ventilated), and to improve precision in our concordance estimates, especially among obese patients.

## Results

Between October and December 2024, 99 adult patients were enrolled (Table 1): 14 healthy volunteers (14.1%), 12 mechanically ventilated patients (12.1%) and 73 non-ventilated patients (73.7%). The cohort was 55.6% male, with a mean age of 54.2 ± 16.3 years and a mean BMI of 27.3 ± 6.4 kg/m^2^.

**Table 1.** Demographic characteristics of patients.

Group	N	Age (mean ± SD)	BMI (mean ± SD)	Male (%)
**Healthy**	14	32 ± 10	24.3 ± 2.1	57%
**Non-ventilated**	73	58 ± 14	27.8 ± 3.0	55%
**Ventilated**	12	61 ± 13	29.5 ± 3.6	58%
**Total**	99	54.2 ± 15.2	27.3 ± 9.5	55.6%

### Overall and Category Concordance

Overall, SCV and THV measurements agreed in 83.8% of cases (83/99; 95% CI 76.4–90.0). By IVC category, concordance was highest for small diameters (< 10 mm) at 93.8 % (15/16; 95% CI 69.8–99.8; p < 0.001); compared to intermediate (10–20 mm) at 84.0% (42/50; 95 % CI 70–90; p < 0.001) and large (> 20 mm) at 82.0% (27/33; 95% CI 77–95; p < 0.00) (Table 2).

**Table 2.** Distribution of concordance by inferior vena cava categories.

Subcostal View Category	10–20 mm	<10 mm	>20 mm	Total	Concordant	Concordance (%)
**10–20mm**	42	2	6	50	42	84.0% (71–92)
**<10mm**	1	15	0	16	15	93.8% (71–99)
**>20mm**	6	0	27	33	27	82% (65–91)

### Subgroup Analyses

Concordance did not differ significantly by ventilation status: non-ventilated 84.1% (95% CI 80.2–93.0) vs. ventilated 83.3% (95% CI 70–90; p = 0.83). Patients with BMI < 30 kg/m^2^ had higher concordance than those with BMI ≥ 30 kg/m^2^ (89.5% [68/76; 95% CI 80.8–95.5] vs. 73.9% [17/23; 95 % CI 51.6–89.8]; p = 0.086). In smaller subgroups, concordance declined further: BMI > 35 kg/m^2^ (n = 11) 72.7% and BMI ≥ 40 kg/m^2^ (n = 5) 60.0 %.

### Agreement (Bland–Altman)

**Figure 4.**
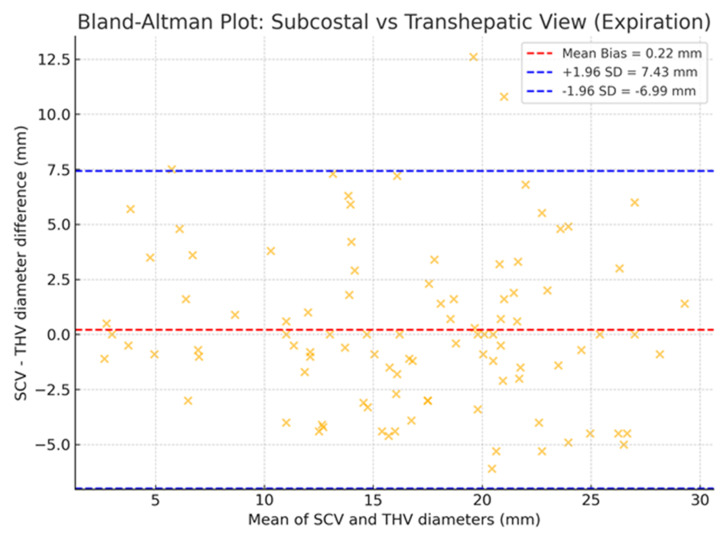
Bland-Altman plot of inferior vena cava (IVC) diameters (n = 99) measured by subcostal views (SCV) and transhepatic views (THV) at end-expiration. Mean bias +0.22 mm; 95% limits of agreement –6.99 mm to +7.43 mm. Only three patients (3%) lay outside these limits.

Bland–Altman analysis showed a mean bias of +0.22 mm between SCV and THV (SCV–THV), indicating minimal systematic difference. The 95% limits of agreement ranged from –6.99 to +7.43 mm, with 97% (96/99) of paired measurements falling within these bounds; only three patients (3%) exceeded these limits (Figure 4).

### Correlation (Pearson)

**Figure 5.**
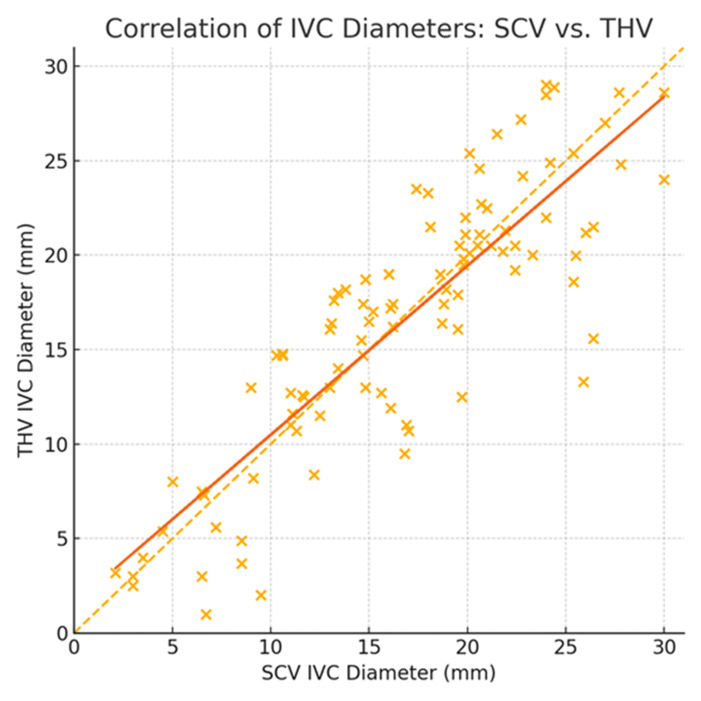
Pearson's correlation coefficient was r = 0.86 (p < 0.001), indicating a strong linear relationship between inferior vena cava (IVC) measurements using subcostal views (SCV) and transthoracic views (THV) overall.

The overall Pearson correlation coefficient was r = 0.86 (p < 0.001), indicating a strong linear relationship (Figure 5). When stratified by category, Pearson's r declined modestly: small IVCs r = 0.81 (n = 16; p < 0.001), intermediate r = 0.78 (n = 50; p < 0.001), and large r = 0.74 (n = 33; p < 0.001).

### Fluid-Responsiveness Assessment

Using a CI-IVC threshold of 42% to define “responders,” THV yielded a sensitivity of 28% and specificity of 93% for predicting fluid responsiveness.

## Discussion

We found overall good concordance between SCV and THV measurements (83.8%). Concordance was highest for small IVCs (93.8%), followed by intermediate (84%) and large (82%) categories. In 16.2% of cases, the two views did not agree. By contrast, the global linear correlation was strong (r = 0.86) but declined within each size category, highlighting the limitation of relying on correlation alone to assess interchangeability, as noted in previous studies [6].

The low mean bias (+0.22 mm) in our Bland–Altman analysis indicated that, on average, SCV and THV measurements are comparable. However, the wide 95% limits of agreement (–6.99 to +7.43 mm) reflect substantial individual variability. Previous studies have reported similar limits (approximately –7.5 to +7.7 mm), confirming that this dispersion is inherent to the THV [7]. Moreover, our overall Pearson correlation coefficient was very strong (r = 0.86), exceeding earlier reports of around 0.73 [8]. When stratified by category, however, correlation declined (small r = 0.81; intermediate r = 0.78; large r = 0.74). This demonstrates that linear association underperforms compared to categorical concordance, remaining high at both extremes (93.8% for small and 82% for large). By asking whether both views agree on the same clinically relevant category, concordance provides a far more practical criterion for the binary decisions. Importantly, no patient was ever classified as small in one view and large in the other, underscoring the robustness of categorical assessment for POCUS-guided decisions.

Anatomical factors help explain why linear correlation between SCV and THV can weaken when the IVC shape departs from circular. With large, circular IVCs (high volume), both views yield similar measurements, but as venous pressure drops the vessel flattens into an asymmetric ellipse, amplifying millimetric discrepancies. Interestingly, our data showed Pearson's r of 0.81 for small IVCs and 0.74 for large IVCs, perhaps because absolute differences between views tend to be smaller when the IVC diameter is low.

Although guidelines endorse both static and dynamic IVC assessments to estimate RAP, categorization on static diameter alone has broad clinical utility when decisions must be made swiftly [3,9,10]. For example, in hemodynamically unstable patients, an IVC diameter <10 mm suggests hypovolemia and likely fluid responsiveness, whereas an IVC diameter >20 mm suggests elevated RAP and possible cardiogenic shock [11]. In dialysis patients, IVC measurement helps prevent intradialytic hypotension [12]. In heart failure, combining IVC size >20 mm with lung ultrasound optimizes diuretic management and reduces rehospitalization [13,14]. In hyponatremia, a small IVC with dry lungs may indicate the need for intravascular volume expansion, whereas a dilated IVC with dry lungs in the setting of acute dyspnea may raise suspicion of pulmonary embolism [15]. For patients with intermediate or small IVCs, fluid tolerance is often assumed, while a large IVC may warrant further evaluation with Venus Excess Ultrasound (VExUS) [17]. Thus, categorical concordance aligns directly with these binary “yes/no” clinical pathways.

Concordance did not differ significantly between ventilated and non-ventilated patients (p = 0.83). Although the decline in concordance from 89.5% (BMI < 30) to 73.9% (BMI ≥ 30) did not reach statistical significance (p = 0.086), there was a clear trend, with further drops to 72.7% (BMI > 35) and 60% (BMI ≥ 40). Given the small numbers in these subgroups, these findings warrant cautious interpretation and future validation. Reduced concordance in obesity likely reflects increased soft-tissue depth degrading image quality especially for deeper THV windows and elevated intra-abdominal pressure altering IVC shape and measurement accuracy [18].

When evaluating responders using a CI-IVC threshold of 42%, concordance between the transhepatic and SCVs was consistent with previous studies [19]. As previously reported, specificity was higher than sensitivity: sensitivity was low (28%, 95% CI: 14–46%), while specificity remained high (93%, 95% CI: 84–98%) [20]. The lower sensitivity of the THV may be explained by anatomical factors: the IVC collapses predominantly in the anteroposterior direction and adopts an elliptical shape during inspiration. This deformation is better captured in SCV. In contrast, the THV has a more lateral insonation angle, which may underestimate collapsibility due to misalignment with the vessel's main axis of collapse [21]. These findings suggest that THV is reliable for confirming fluid responsiveness when high collapsibility is observed but may underestimate the identification of true responders.

Limitations include the lack of formal interobserver reproducibility testing. In our study, each patient was scanned by a single operator.

## Conclusion

Categorical evaluation of the IVC using the THV is a viable alternative to the subcostal approach when the SCV is not feasible. This method supports rapid, clinically meaningful decision-making in critically ill patients and maximizes the applicability of POCUS. Nevertheless, its utility in patients with elevated BMI may be limited, and further research is warranted to explore this subgroup.
